# Formulation of zein nanoparticles for augmenting the anti-inflammatory activity of dexketoprofen

**DOI:** 10.3389/fphar.2025.1560585

**Published:** 2025-07-01

**Authors:** Mahmoud A. El Hassab, Mona H. Ibrahim, Sherif S. Abdel Mageed, Abdulla M. A. Mahmoud, Zainab Sabry Othman Ahmed, Shaimaa Mosallam, Manar Adel Abdelbari, Eman F. Khaleel, Maram A. El Hasab, Asmaa Elsayyad, Shady Allam, Moaz A. Eltabeeb, Ahmed T. Negmeldin, Wagdy M. Eldehna, Rofida Albash

**Affiliations:** ^1^ Department of Medicinal Chemistry, Faculty of Pharmacy, King Salaman International University (KSIU), South Sinai, Egypt; ^2^ Department of Pharmaceutical Medicinal Chemistry and Drug Design, Faculty of Pharmacy (Girls), Al-Azhar University, Cairo, Egypt; ^3^ Pharmacology and Toxicology Department, Faculty of Pharmacy, Badr University in Cairo (BUC), Cairo, Egypt; ^4^ Department of Cytology and Histology, Faculty of Veterinary Medicine, Cairo University, Giza, Egypt; ^5^ Faculty of Veterinary Medicine, King Salman International University, South Sinai, Egypt; ^6^ Department of Pharmaceutics and Industrial Pharmacy, Faculty of Pharmacy, October 6 University, Giza, Egypt; ^7^ Department of Medical Physiology, College of Medicine, King Khalid University, Asir, Saudi Arabia; ^8^ Department of Pediatrics, Faculty of Medicine, Tanta University, Tanta, Egypt; ^9^ Department of Pharmacology, Faculty of Veterinary Medicine, Mansoura University, Mansoura, Egypt; ^10^ Department of Physiology, and Pharmacology, Faculty of Veterinary Medicine, King Salman International University, South Sinai, Egypt; ^11^ Department of Pharmacology and Toxicology, Faculty of Pharmacy, Menoufia University, Menoufia, Egypt; ^12^ Department of Pharmacology and Toxicology, Faculty of Pharmacy, Menoufia National University, Menoufia, Egypt; ^13^ Department of Industrial Pharmacy, College of Pharmaceutical Sciences and Drug Manufacturing, Misr University for Science and Technology, Giza, Egypt; ^14^ Department of Pharmaceutical Sciences, College of Pharmacy and Thumbay Research Institute for Precision Medicine, Gulf Medical University, Ajman, United Arab Emirates; ^15^ Department of Pharmaceutical Organic Chemistry, Faculty of Pharmacy, Cairo University, Cairo, Egypt; ^16^ Department of Pharmaceutical Chemistry, Faculty of Pharmacy, Kafrelsheikh University, Kafrelsheikh, Egypt; ^17^ Department of Pharmaceutics, College of Pharmaceutical Sciences and Drug Manufacturing, Misr University for Science and Technology, Giza, Egypt

**Keywords:** dexketoprofen, zein nanoparticles, inflammation, formalin-induced paw edema, topical delivery, docking

## Abstract

**Introduction:**

Topical delivery of anti-inflammatory drugs is an important strategy for enhancing therapeutic efficacy while minimizing systemic side effects. This study focuses on improving the anti-inflammatory activity of Dexketoprofen by developing zein nanoparticles (ZNs) as a novel topical carrier system, aiming to optimize drug delivery and patient outcomes.

**Methods:**

Dexketoprofen-loaded ZNs were prepared using an ethanol injection technique and optimized via a 2^3^ full factorial design. The effects of three variables—phosphatidylcholine (PC) amount (X_1_), type of surface-active agent (SAA, X_2_), and SAA amount (X_3_)—were evaluated on entrapment efficiency (EE%), particle size (PS), polydispersity index (PDI), and zeta potential (ZP). Design-Expert® software was employed to identify the optimal formulation. Additionally, molecular docking studies were performed to explore interactions between Dexketoprofen and formulation components. The selected formulation (F7) was further characterized for morphology using scanning electron microscopy. *In vivo* efficacy was assessed using a formalin-induced paw edema model in rats, and histopathological analysis was conducted to evaluate skin irritation potential.

**Results:**

The optimal formulation (F7), prepared with 200 mg PC and 20 mg Pluronic F127, demonstrated an entrapment efficiency of 92.44 ± 7.21%, particle size of 91.88 ± 3.01 nm, PDI of 0.42 ± 0.02, and zeta potential of −24.10 ± 0.29 mV. F7 exhibited a smooth, spherical morphology. *In vivo* studies revealed significantly enhanced anti-inflammatory activity compared to free Dexketoprofen. Histopathological examination confirmed the non-irritant nature of the formulated ZNs on rat skin.

**Discussion:**

These findings highlight the effectiveness of zein nanoparticles as a promising topical delivery system for Dexketoprofen. The optimized ZNs not only improved drug entrapment and stability but also provided superior anti-inflammatory efficacy and excellent skin tolerability, suggesting their potential for the treatment of inflammatory skin conditions.

## Introduction

Nanoparticles are colloidal drug carriers made of natural or synthetic polymers (Albash et al., 2025). They have demonstrated considerable interest in drug delivery (Mitchell et al., 2021). Different nanoparticles have been prepared to improve the therapeutic and pharmacological properties of free drugs, including instability (Yetisgin et al., 2020), serious side effects, low bioavailability, and reduced aqueous solubility, and some of them can improve the absence of targeted delivery (Chenthamara et al., 2019). They have high drug-loading capacity and can encapsulate different drugs with different solubilities. Protein can be used to prepare nanoparticles as it can provide many advantages, such as environmental tolerance, wide availability, biodegradability, and high drug-binding capacity (Hong et al., 2020). Proteins may be animal-derived, such as elastin, gelatin, albumin, casein, lysozyme, and lactoferrin (Kianfar, 2021), or plant-derived, such as zein, soy protein isolate, glutenin, gliadin, and walnut protein isolate (Liu et al., 2023). Plant-derived proteins are cheaper, decrease the risk of spreading diseases, and are less immunogenic (Reddy and Rapisarda, 2021).

Zein is the main corn storage protein (Zhang et al., 2021), which contains 44%–79% of the endosperm protein. It is widely available in nature and can be extracted from corn easily. Zein is soluble in aqueous ethanol and aqueous acetone and insoluble in water and absolute ethanol (Wu et al., 2023). It is also a prolamin and has four classes (α, β, γ, and δ) with different molecular sizes, solubility, and peptide chains. The richest constituent is α-zein, containing approximately 80% of the whole zein, followed by γ-zein, which contains approximately 15%. The available zein is commercially presented in yellow and white forms. Yellow zein is 90% pure and contains xanthophyll pigments in a high concentration, while white zein has higher purity with a minor amount of xanthophylls (De Marco, 2022). Zein has the advantage of being non-toxic, economical, biodegradable, and biocompatible (Radwan et al., 2020). Furthermore, previous studies included zein nanoparticles for the delivery of anti-inflammatory drugs, such as quercetin and resveratrol (Penalva et al., 2017; Liu et al., 2021). Zein has attracted growing attention as a feasible material for drug delivery systems based on nanoparticles because of its amphiphilic nature and biocompatibility, which make it particularly suitable for carrier applications.

Dexketoprofen is a non-steroidal anti-inflammatory propionic acid derivative with the ability to treat acute and chronic pain and inflammation (Moore and Barden, 2008). It has antipyretic, anti-inflammatory, and analgesic effects. Its action is caused by the inhibition of cyclooxygenase-1 (Cox-1), which is involved in the production of prostaglandins with physiological functions, and cyclooxygenase-2 (Cox-2), which facilitates the synthesis of pro-inflammatory prostaglandins at the inflammation site (Kuczyńska et al., 2022). Dexketoprofen inhibits prostaglandin synthesis strongly. It is a ketoprofen (S+) enantiomer but with a stronger effect and fewer serious adverse effects. It is effective at low doses and well tolerated, making it a readily used preparation (Gaskell et al., 2017). Dexketoprofen has a melting point of 104.94°C and Log P = 2.65, which indicates ambient lipophilicity, making the drug perfect for topical administration (Carpenter et al., 2024).

Inflammation is a protective approach in response to tissue injury, microbial infection, or other detrimental conditions (Soliman et al., 2023; Abdelbari et al., 2023). The typical symptoms of inflammation include pain, redness, swelling, and heat (Ahmed, 2011). Edema is a condition characterized by fluid accumulation resulting from an abnormal increase in interstitial fluid volume, typically due to inflammation (Trayes et al., 2013).

The aim of this study was to prepare zein nanoparticles (ZNs) loaded with dexketoprofen for the effective management of inflammation. ZNs are biocompatible and have the capacity to encapsulate both hydrophilic and hydrophobic drugs with good stability (Elmahboub et al., 2024). Dexketoprofen-loaded ZNs were prepared using Pluronic F127 (PF127) or Pluronic F86 (PF86) as a surface-active agent (SAA) to improve the drug’s penetration. For the optimization of the prepared ZNs, a 2^3^ full-factorial design was applied to assess the influence of different variables on the characterization of the prepared ZN formulations and determine the best formulation. Phosphatidylcholine (PC) amount (mg) (X_1_), type of SAA (X_2_), and SAA amount (mg) (X_3_) were chosen as independent variables, while the responses were entrapment efficiency (EE%) (Y_1_), particle size (PS) (Y_2_), polydispersity index (PDI) (Y_3_), and zeta potential (ZP) (Y_4_). The optimal ZN morphology was investigated. Furthermore, this is the first report on the *in vivo* anti-inflammatory potential of the optimal dexketoprofen-loaded ZN formulation against formalin-induced paw edema. Safety assessment was also evaluated using a histopathological study.

## Materials

Dexketoprofen was sourced from Utopia Pharmaceuticals, Cairo, Egypt; formalin, zein (90% purity), PF127, and PF86 were purchased from Sigma-Aldrich, MO, United States. All chemicals used were of the highest available analytical quality.

## Methods

### Fabrication of dexketoprofen-loaded ZNs

ZNs were synthesized using an ethanol injection method with slight modifications (Abdelbari et al., 2021). Initially, zein (10 mg) and PC with surfactants at varying concentrations were measured and dissolved in 2 mL of ethanol (95%) (1:5, v/v%). Dexketoprofen (50 mg) was dissolved in 10 mL of distilled water, and the resulting solution was injected into the lipophilic mixture and stirred using a magnetic stirrer (Model MSH-20D, GmbH, Berlin, Germany) at 1,500 rpm for 30 min at room temperature to ensure complete solvent evaporation. A probe sonicator (JY-92-II, Xinzhi, China) was used to enhance the homogeneity of the particles in the dispersion medium of ZNs, with sonication conducted at 40% amplitude for 5 min, consisting of 3 s on and 3 s off. Finally, the formulations were stored in a refrigerator for maturity.

### *In vitro* characterization of dexketoprofen-loaded ZNs

#### EE %

The percentage of dexketoprofen encapsulated in ZNs was assessed using a spectrophotometer. Cooling ultracentrifuge (Sigma 3 K 30, Germany) was used for 1 hour at 4°C and 20,000 rpm (Adel et al., 2023). The precipitate was then lysed using 10 mL methanol at room temperature, and 1 mL of the solution was measured using an ultraviolet spectrophotometer at λ_max_ 242 nm (Carpenter et al., 2024) (Shimadzu UV1650 Spectrophotometer, Kyoto, Japan). The drug’s EE% was calculated thrice using Equation 1:

$$\text{EE }\left(\%\right)=\frac{\text{Total}\;\text{amount}\;\text{of}\;d\text{exketoprofen}-\text{Free}\;d\text{exketoprofen}}{\text{Total}\;\text{amount}\;\text{of}\;d\text{exketoprofen}}\times 100.$$ (1)

The average PS and PDI of the prepared ZNs were measured using a Zetasizer (Malvern Instrument Ltd., United Kingdom), applying the light-scattering procedure to the ZN suspension in distilled water (Abdelbari et al., 2024). Using the same Zetasizer, the electrophoretic mobility of the prepared ZNs was measured to determine the ZP. All measurements were performed in triplets (Mosallam et al., 2021a).

#### Experimental design and optimization

The influence of the variables on the characterization of the prepared ZNs was determined using a 2^3^ full-factorial design with Design-Expert^®^ software version 11 (Stat-Ease, Inc., Minneapolis, MN, United States). The investigated variables were PC amount (mg) (X_1_), type of SAA (X_2_), and SAA amount (mg) (X_3_), each variable with two levels, as displayed in Table 1. An analysis of variance (ANOVA) test was used to evaluate parameters and determine the optimal formulation. *P* ≤ 0.05 reflected a significant difference.

**TABLE 1:** Variables and levels used in the preparation of dexketoprofen-loaded ZNs using a (2^3^) full-factorial design and the statistical analysis summary of the full-factorial design (2^3^) used for the optimization of the prepared ZNs.

Studied variable	Level	
X_1_: PC amount (mg)	100	200
X_2_: Type of SAA	PF127	PF86
X_3_: SAA amount (mg)	10	20
Response	Desirability constraints	
Y_1_: EE%	Maximize	
Y_2_: PS (nm)	Minimize	
Y_3_: PDI	Minimize	
Y_4_: ZP (mV)	Maximize (as absolute value)	

#### Docking studies

The docking studies were assessed using molecular operating environment (MOE) software version 2019.0102 (Hassab et al., 2021). The compounds were drawn using ChemDraw 20.1.1 and transformed using MOE to three-dimensional (3D) structures. The energy of the four compounds (dexketoprofen, lecithin, zein, and PF127) was minimized using AMBER10:EHT before initiating the docking. Each component (lecithin, zein, and PF127) was solvated in distilled water and then used as a potential receptor to bind with dexketoprofen. The docking studies were directed at maximum accuracy using induced fit protocol and triangle matcher as the scoring function. The results were examined based on docking scores and molecular interactions.

### Characterization of the optimal dexketoprofen-loaded ZNs

#### Determination of the amount of drug release

USP dissolution apparatus II (Pharma Test, Hainburg, Germany) was used to assess *in vitro* drug release for 12 h at 37°C. An amount of 2 mL of the optimal ZN formulation containing 5 mg of dexketoprofen was put into tubes with a permeation area (3.14 cm^2^), with one end firmly sealed with a cellulose membrane and the other end linked to the shaft of the dissolution equipment rather than the baskets. The receptor medium was 50 mL of phosphate buffer saline solution (pH 7.4). Aliquots were withdrawn at different time intervals up to 12 h. The samples were examined using a UV spectrophotometer set to λ_max_ = 242 nm. The measurements were carried out in triplicate.

#### Stability study

The optimal ZN formulation was stored at 4°C for 3 months. The initial measurements were compared with results obtained after storage to evaluate physical stability. The EE%, PS, PDI, ZP, and amount of drug released after 6 h (Q6h; %) from the nanoparticles were measured. Statistical significance was analyzed using Student’s t-test in SPSS^®^ software 22.0. A difference at *p* ≤ 0.05 was considered significant.

### *In vivo* studies

#### Experimental animals

Male Wistar albino rats, aged 7 weeks and weighing from 200 to 250 g, were obtained from the animal house at the Faculty of Pharmacy, Badr University, Cairo, Egypt. The rats were provided with unrestricted access to standard rat food and water in a controlled environment (23°C ± 1°C and 40%–60% humidity). After 1 week of acclimatization, the experiment was conducted in accordance with NIH guidelines (NIH Publication No. 85–23, revised 2011) for the care and use of animals, and it was accepted by the research ethics committee of the Faculty of Pharmacy, Badr University, Cairo (approval number, IACUC/PHA/162/A/2024).

#### Animal groups

Rats were randomly assigned to four groups, each consisting of six animals, and were labeled as follows:

• Group I: control group (CTRL).
• Group II: paw edema group (PE).
• Group III: standard group (DKP).
• Group IV: dexketoprofen-loaded ZN formulation group (NanoDKP).

During the experiment, rats were pre-treated with a single oral dose of dexketoprofen-loaded ZN formulation (0.1 mg/kg, p.o.) for Group IV (Soyocak et al., 2019). The control group received the vehicle orally at a matched dose volume of 1 mL/kg, and the standard group received dexketoprofen (0.1 mg/kg, p.o.) (Soyocak et al., 2019). One hour after the final administration of the test drug or vehicle, all rats in groups II, III, and IV were given a sub-plantar injection of 0.1 mL of 5% formalin to induce edema in the left paw (Abdel Mageed et al., 2022; Bancroft and Gamble, 2008). To ensure dose equivalence, the dose of dexketoprofen-loaded Zn nanoparticles administered to Group IV was calculated to deliver an amount of dexketoprofen equivalent to 0.1 mg/kg body weight (p.o.), consistent with the standard group.

#### Formalin-induced paw edema in rats

Formalin-induced edema was induced in the left hind paw of rats in all groups via a sub-plantar injection of 0.1 mL of 5% formalin, as previously described (Abdel Mageed et al., 2022; Bancroft and Gamble, 2008), except for the control group, which received normal saline. All groups received their respective treatments 1 h prior to the formalin injection. Paw thickness was measured immediately after the formalin injection (time 0) and at 1, 2, 4, and 24 h using a Vernier caliper to assess the degree of inflammation. At the end of the experiment, rats were euthanized under anesthesia using thiopental (50 mg/kg, i.p) (Mostafa et al., 2023); their paws were removed, and blood samples were collected.

#### Evaluation of oxidative stress and inflammatory markers

##### ELISA technique

In compliance with the manufacturer’s instructions, the ELISA technique was conducted using the Thermo Scientific Multiskan FC Microplate Reader (Thermo Fisher Scientific, United States) to estimate myeloperoxidase (MPO) (Cat#: E4581-100; Bio Vision Incorporated, Milpitas, CA, United States), Cox-2 (Cat#: A74115, antibodies, United Kingdom), and interleukin 6 (IL-6) (Cat#: MBS269892, MyBioSource, United States) in tissue homogenate, whereas tumor necrosis factor-α (TNF-α) (Cat#: MBS2507393; MyBioSource, United States) and prostaglandin E2 (PGE2) (Cat#: ab287802, Abcam, United Kingdom) were estimated in the serum sample.

##### Colorimetric analysis

Malondialdehyde (MDA) (Abcam, Cat# ab118970) and reduced glutathione (GSH) (Novus Biologicals, Cat# NBP3-25794) were assessed in tissue homogenate using colorimetric reagents, in compliance with the manufacturer’s instructions, using a UV–Visible spectrophotometer (Shimadzu UV-1800, Japan).

##### *In vivo* histopathological examination

Skin tissue from all groups was dissected, fixed in 10% neutral buffered formalin (10% NBF) for 48 h, washed, and embedded for dehydration in ascending ethanol grades. The dehydrated sections were cleared in xylene, embedded in paraffin wax, sectioned at 3–4 µm thickness, deparaffinized, and stained with hematoxylin and eosin (H&E) stain (Bancroft and Gamble, 2008). The stained slides were inspected using a light microscope (Leica DM500), and the images were captured using a camera (Leica ICC50 HD) attached to the microscope and examined using image analysis software [Leica Microsystems, LAS version 3.8.0 (Build: 878); Leica image analyzer computer system] at the Light Microscopy Unit, Cairo University Research Park (CURP), Faculty of Agriculture, Cairo University, Giza, Egypt.

##### Statistical analysis

The data were presented as the mean ± standard deviation (SD) and analyzed using one-way ANOVA to investigate group differences, followed by Tukey’s *post hoc* test for comparison. Results reflected a significant difference if *p* ≤ 0.05. All statistical analyses were executed using GraphPad Prism version 8 (San Diego, California, United States).

## Results and discussion

### Factorial design outcomes

A 2^3^ full-factorial design involved eight formulations (F1–F8). All the measured responses are displayed in Table 1. Adequate precision is the signal-to-noise ratio (Mostafa et al., 2023) that reflects the model’s ability to navigate the design space. It is preferable for this value to exceed 4 (Abdelbary et al., 2016), and this was achieved in all responses, as displayed in Table 1. The predicted *R* ^2^ also affects the models’ quality (Abdelbari et al., 2021). It is necessary that the adjusted and predicted *R* ^2^ show an acceptable agreement (Al-mahallawi et al., 2015), which is displayed in all the response outcomes (Table 1).

### Influence of variables on EE%

**FIGURE 1:**
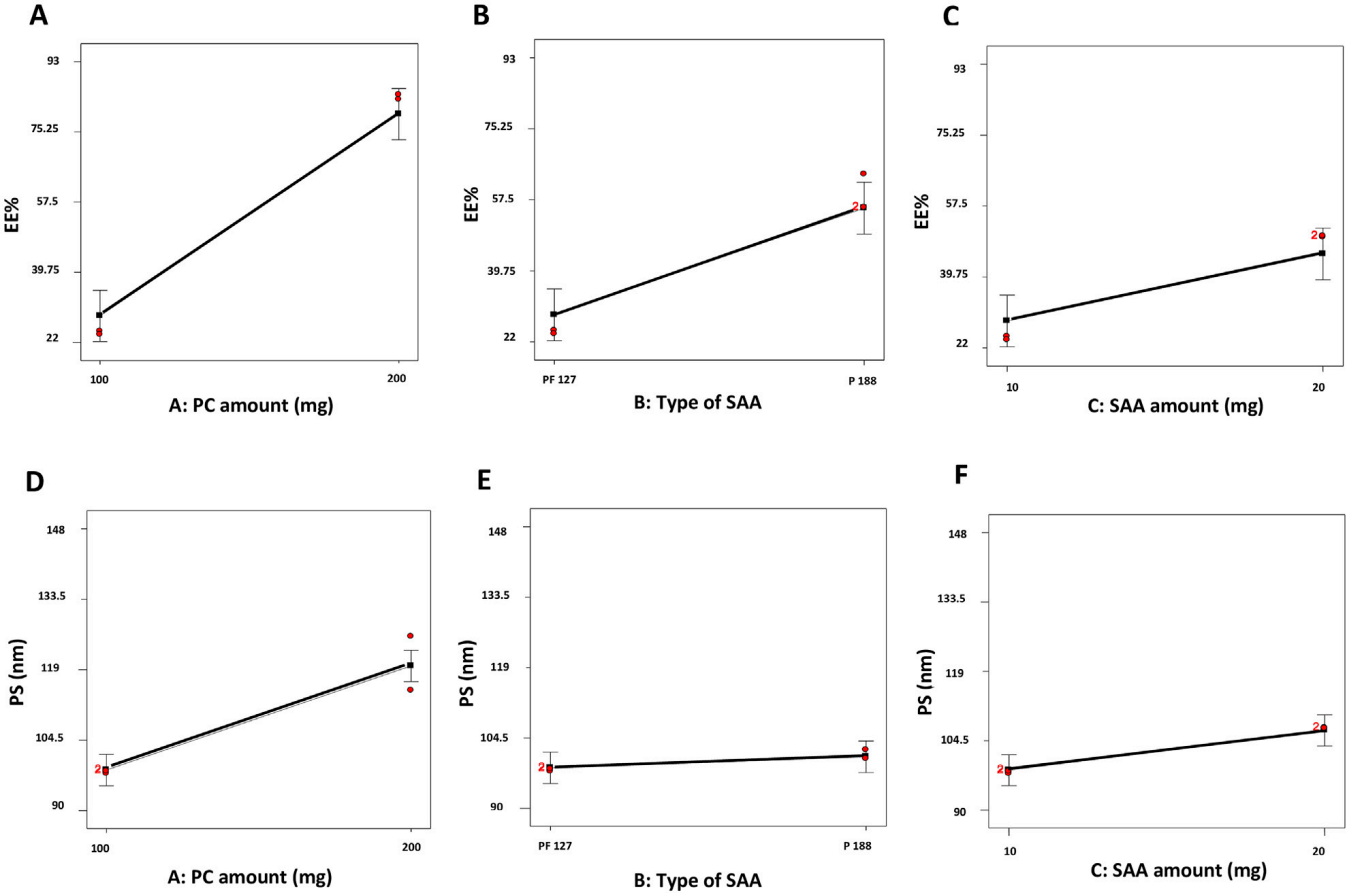
Linear plots demonstrating the effect of the PC amount (mg) (X_1_), type of SAA (X_2_), and SAA amount (mg) (X_3_) on EE% **(A–C)** and PS **(D–F)** of dexketoprofen-loaded ZNs. PC, phosphatidylcholine; EE%, encapsulation efficiency percent; PS, particle size; ZNs, zein nanoparticles.

The EE% of dexketoprofen in the prepared ZNs ranged from 23.16% ± 0.54% to 92.44% ± 7.21% (Table 2). The influences of PC amount (mg) (X_1_), type of SAA (X_2_), and SAA amount (mg) (X_3_) on EE% are demonstrated as linear plots in Figures 1A–C. Statistical analysis of the data revealed that the three variables significantly influence the EE% (*p* < 0.05).

**TABLE 2:** Results of ZN formulations of the complete (2^3^) full -factorial design.

Formula	X_1_ PC amount (mg)	X_2_ Type of SAA	X_3_ SAA amount (mg)	Y_1_ EE%	Y_2_ PS (nm)	Y_3_ PDI	Y_4_ ZP (mV)
F1	100	PF 127	10	23.16 ± 0.54	97.19 ± 1.13	0.32 ± 0.02	−22.40 ± 0.61
F2	100	P 188	10	63.89 ± 8.33	101.10 ± 0.90	0.24 ± 0.02	−14.00 ± 1.39
F3	100	PF 127	20	50.09 ± 0.44	106.40 ± 1.24	0.40 ± 0.01	−24.20 ± 0.26
F4	100	P 188	20	55.04 ± 7.07	95.69 ± 7.74	0.58 ± 0.07	−27.20 ± 0.30
F5	200	PF 127	10	90.24 ± 5.07	121.00 ± 5.71	0.65 ± 0.05	−11.40 ± 0.67
F6	200	P 188	10	78.13 ± 3.31	147.20 ± 16.54	0.79 ± 0.19	−23.70 ± 1.71
F7	200	PF 127	20	92.44 ± 7.21	91.88 ± 3.01	0.42 ± 0.02	−24.10 ± 0.29
F8	200	P 188	20	90.66 ± 1.54	107.70 ± 0.87	0.42 ± 0.01	−19.70 ± 0.91

All the prepared ZNs contained equal amount of dexketoprofen (50 mg).

Data are presented as the mean ± SD (n = 3).

First, increasing the PC amount (mg) (X_1_) significantly improved the EE% of the prepared ZNs (Tefas et al., 2017); this might have resulted from the hydrophobic interactions with the PC bilayer structure (Were et al., 2003), which decreases the membrane’s fluidity, therefore increasing the drug’s EE% in the prepared ZNs (Albash et al., 2024). Additionally, a higher PC amount increases viscosity, which might reduce the external diffusion of dexketoprofen (Albash et al., 2021).

Next, the type of SAA (X_2_) had a significant influence on the prepared ZNs’ EE% as those prepared using PF86 had a higher EE% than those prepared using PF127. This result is attributed to the surfactant PF86, which helps stabilize and solubilize the drug molecule, allowing it to be encapsulated within the lipid matrix and at the surface of ZNs (Ali et al., 2023). This result can also be explained through the HLB values of both SAAs, which are 22 and 29 for PF127 and PF86, respectively (Mostafa et al., 2023; Abdelbary et al., 2016); as the drug is hydrophilic, it had a higher EE% when combined with PF86, which has a higher HLB value and, consequently, lower lipophilicity (Albash et al., 2021).

Finally, the SAA amount (mg) (X_3_) also had a significant influence on EE% as increasing the SAA amount (mg) increased the EE% of the prepared ZNs, which can be attributed to the effect of the high SAA amount in forming a layer that enhances the vesicle interface stability, offering more room inside ZNs to encapsulate more drug (Abdelbari et al., 2021).

### Influence of variables on PS

All the prepared ZNs had low PS, which ranged from 91.88 ± 3.01 to 147.20 ± 16.54 nm (Table 2). Low PS ensures the production of a stable system and enhances skin penetration (Roberts et al., 2017). The influences of PC amount (mg) (X_1_), type of SAA (X_2_), and SAA amount (mg) (X_3_) on PS are demonstrated as linear plots in Figures 1D–F. Statistical analysis of the data revealed that the three variables significantly influence the PS (*p* < 0.05).

Increasing the PC amount (mg) (X_1_) significantly increased PS. This result agrees with the EE% results, where increasing the PC amount (mg) resulted in a higher EE% (Albash et al., 2021). Moreover, the type of SAA significantly affected the PS of the prepared ZNs as those prepared using PF86 had a larger PS than those prepared using PF127. The higher amount of dexketoprofen encapsulated within the prepared ZNs might be the reason behind increasing their PS (Mosallam et al., 2021b). Finally, the SAA amount (mg) (X_3_) also had a significant influence on the PS (Rania et al., 2024).

### Influence of variables on PDI

**FIGURE 2:**
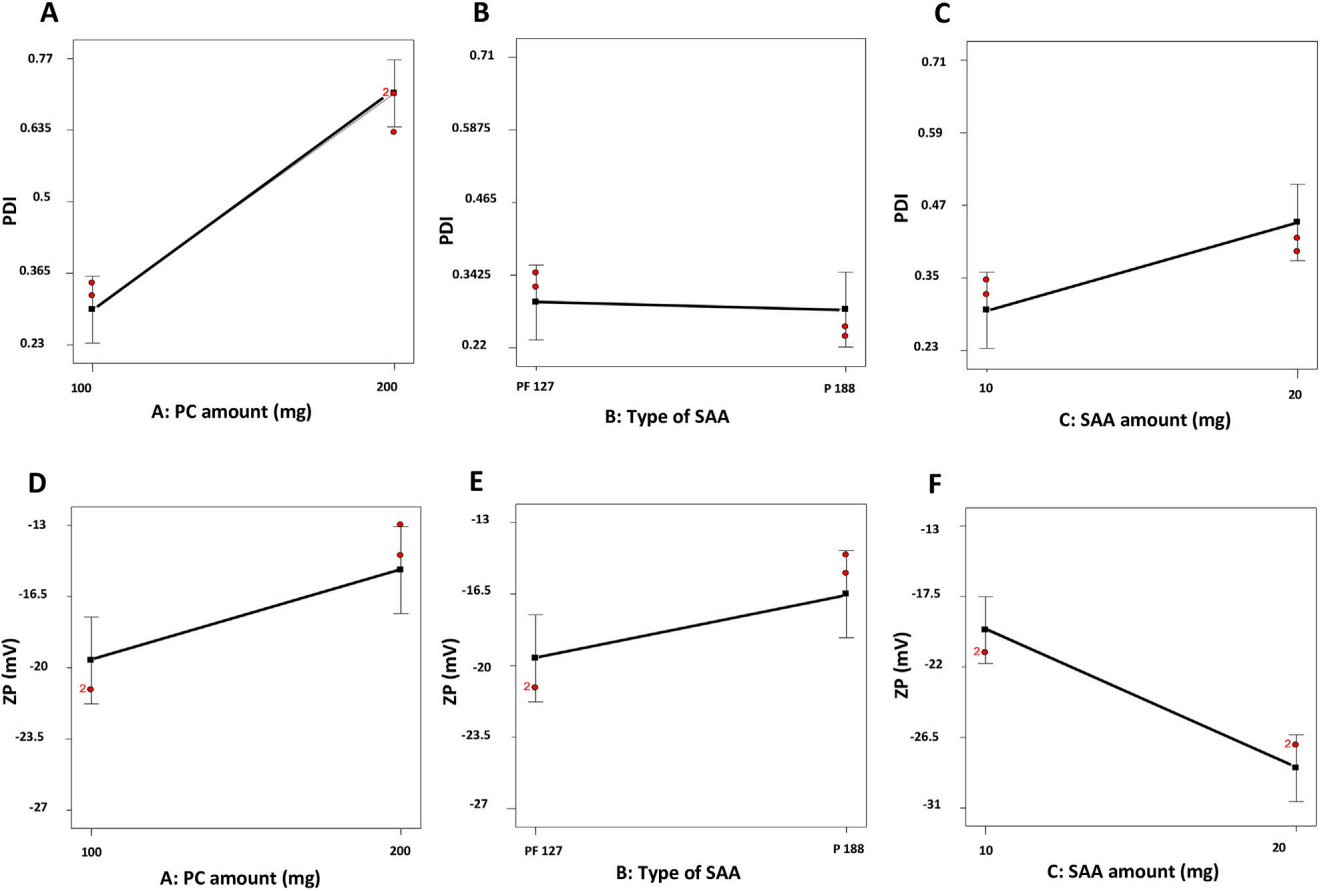
Linear plots demonstrating the effect of the PC amount (mg) (X_1_), type of SAA (X_2_), and SAA amount (mg) (X_3_) on PDI **(A–C)** and ZP **(D–F)** of dexketoprofen-loaded ZNs. PC, phosphatidylcholine; PDI, polydispersity index; ZP, zeta potential; ZNs, zein nanoparticles.

The PDI reflects the system’s heterogeneity, and it occurs between 0 and 1 (Soliman S et al., 2022). The PDI of the prepared ZNs ranged between 0.24 ± 0.02 and 0.79 ± 0.19 (Table 2), reflecting that some of the prepared formulations are polydisperse. Factorial analysis showed that PC amount (mg) (X_1_) significantly influenced the PDI (*p* < 0.05). Increasing the PC amount significantly increased the PDI values, probably due to the high PC amount that reached maximum coverage of the surface of the prepared ZNs, which formed a superficial adsorption layer producing steric resistance (Wang et al., 2019). However, both types of SAA (X_2_) and SAA amount (mg) (X_3_) had a non-significant influence on the PDI of the prepared ZNs. Results are demonstrated as linear plots in Figures 2A–C.

### Influence of variables on ZP

The stability of a system can be evaluated through the ZP; it is a value that reflects the total charges gained by the particles. A ZP value between ±30 mV reflects a stable system as it indicates a sufficient electric repulsive force between particles (Salama et al., 2022). ZP values of the prepared ZNs ranged from −11.40 ± 0.67 to −27.20 ± 0.30 mV (Table 2), which indicates the high stability of the formulated ZNs. Factorial analysis exposed that the SAA amount (mg) (X_3_) had a significant influence on ZP (*p* < 0.05). As shown in Figures 2D–F, increasing the SAA amount increased the ZP as an absolute value. Neither the PC amount (mg) (X1) nor the type of SAA (X2) had a significant influence on the ZP of the prepared ZNs.

### Determination of the optimal dexketoprofen-loaded ZNs

Design-Expert^®^ software was used to select the optimal ZN formulation by evaluating the results statistically. The optimal formulation had the highest EE% and most stable ZP and the lowest PS and PDI. F7 was selected as the optimal formulation, with a desirability of 0.787. F7 was prepared using 10 mg zein, 200 mg PC, and 20 mg PF127 and had an EE% of 92.44% ± 7.21%, a PS of 91.88 ± 3.01 nm, a PDI of 0.42 ± 0.02, and a ZP of −24.10 ± 0.29 mV.

### Docking studies

**FIGURE 3:**
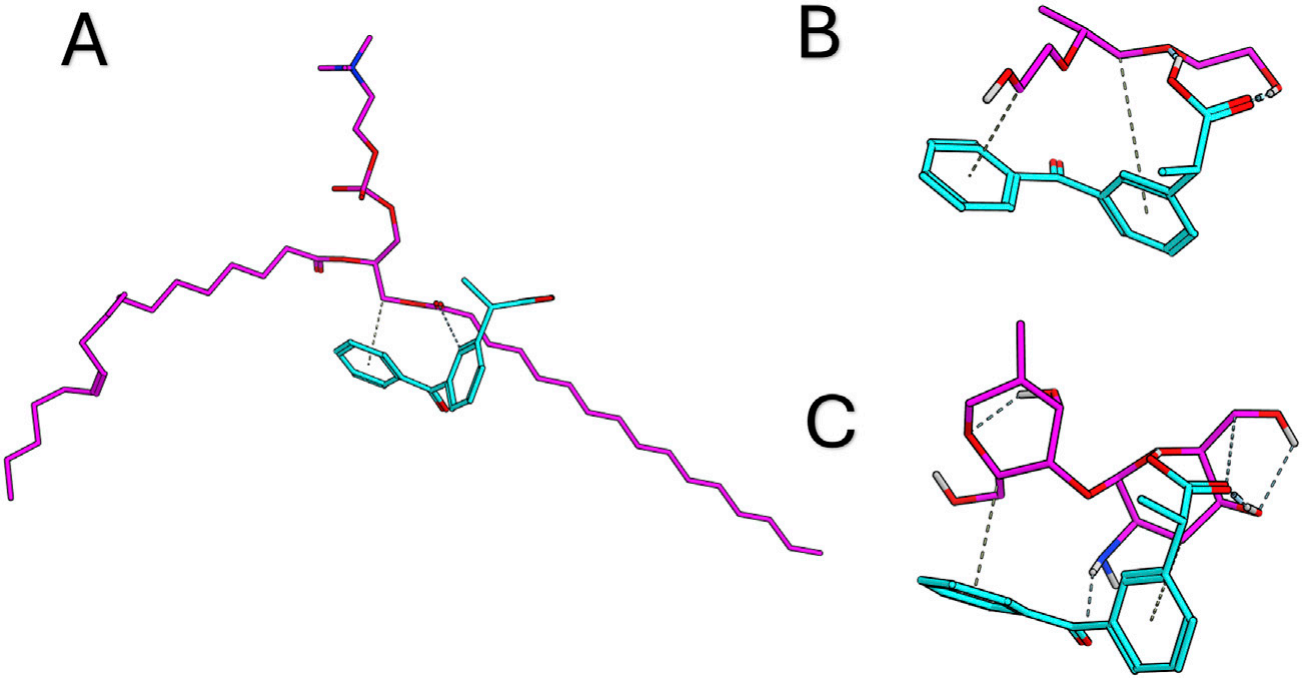
Docking results for dexketoprofen with the formulation components and the 3D interaction diagram between dexketoprofen, **(A)** lecithin, **(B)** Pluronic F127, and **(C)** zein.

The study was conducted to explore the potential interactions between dexketoprofen and the components of the formula. Each component was assessed for its ability to bind with dexketoprofen. Notably, dexketoprofen showed favorable binding interactions with all three components of the formulation. The recorded binding affinities were −5.8, −6.6, and −5.3 kcal/mol for PC, zein, and Pluronic F127, respectively. As illustrated in Figure 3, dexketoprofen is involved in multiple interactions with these components. The docking analysis particularly highlighted the strong binding affinity of zein for dexketoprofen, suggesting that incorporating zein into the formulation may be essential.

### Characterization of the optimal dexketoprofen-loaded ZNs

#### *In vitro* release profile

**FIGURE 4:**
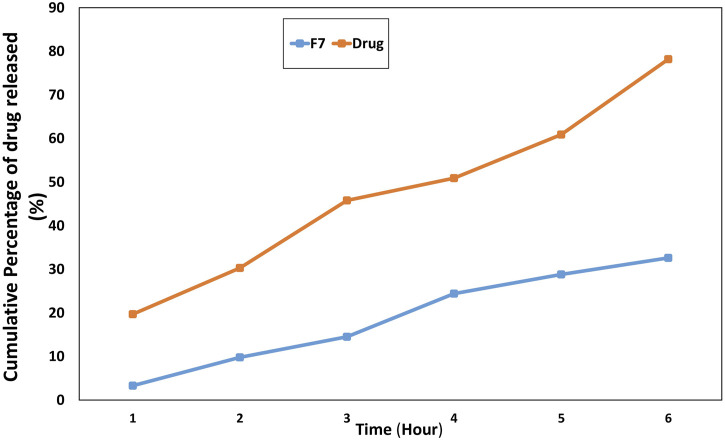
*In vitro* release behavior of F7 compared with the drug.

The adverse effects can be reduced in the modified release manner as the drug exists at the target site for an extended time (Sankhyan and Pawar, 2013). F7 exhibited sustained *in vitro* release behavior of the drug, with approximately 78% of the drug released within 12 h, compared to approximately 100% from the drug solution, as shown in Figure 4. The presence of the hydrophobic carrier zein played an essential role in obtaining a modified release manner (Franco and De Marco, 2020).

#### Stability study

The results revealed that F7 exhibited respectable storage stability. The stored F7 showed non-significant deviations in appearance, EE%, PS, PDI, and ZP (*p* > 0.05) compared to the fresh F7 (Table 3).

**TABLE 3:** Stability study.

Parameter	Fresh formula	Stored formula
EE%	92.44 ± 7.21	93.13 ± 4.51
PS (nm)	91.88 ± 3.01	94.91 ± 2.03
PDI	0.42 ± 0.02	0.45 ± 0.12
ZP (mV)	−24.10 ± 0.29	−23.10 ± 0.58

### *In vivo* studies (effect of F7)

**FIGURE 5:**
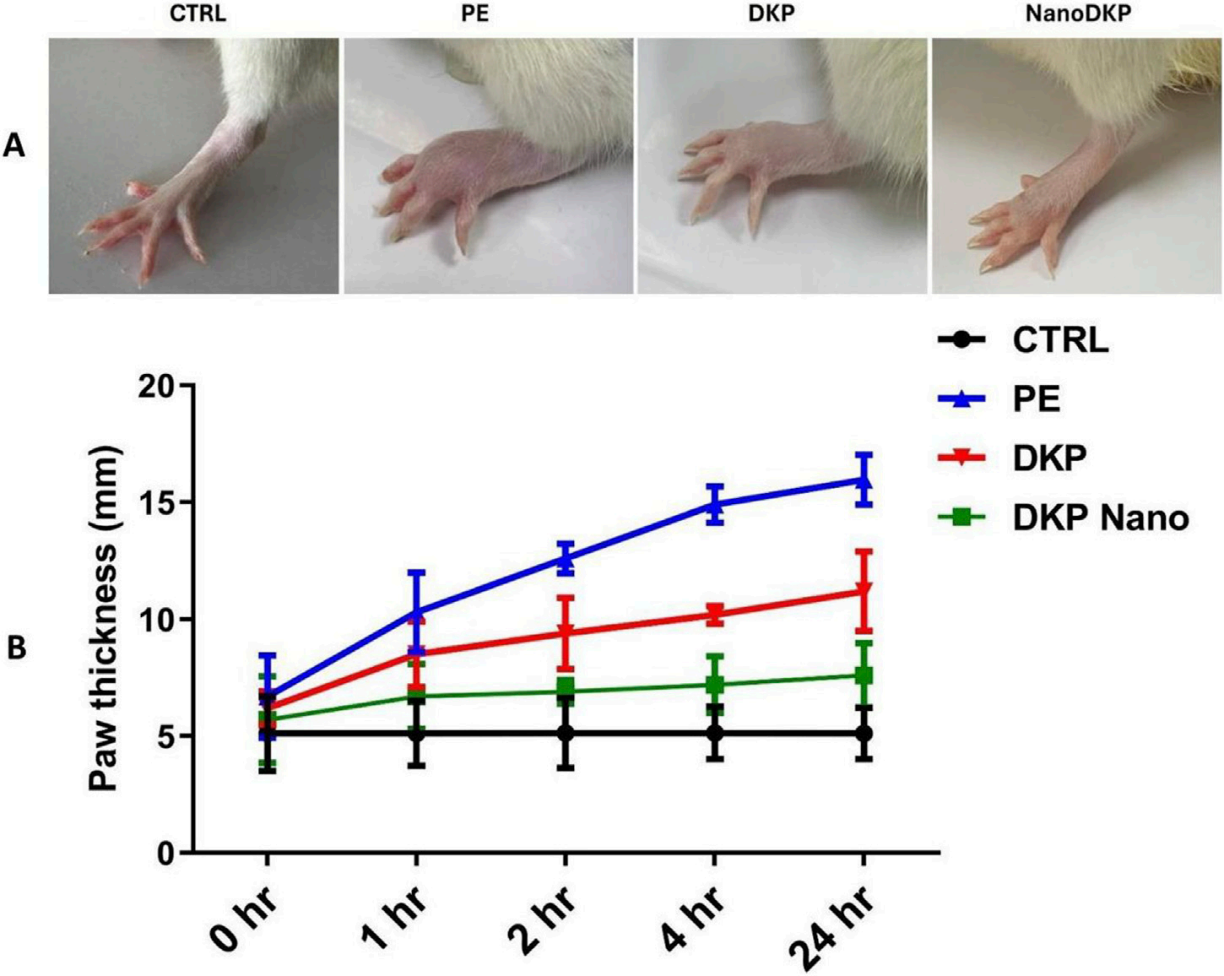
Effect of the optimal ZN formulation (F7) on formalin-induced paw edema in rats. **(A)** Representative images show foot swelling in each group 24 h after edema induction. **(B)** Change in paw thickness (mm) in the experimental rats. Edema was induced by injecting 0.1 mL of a 5% formalin solution into the sub-plantar surface of the left-hind paw. The mean values ±SD for six rats per group (n = 6) are shown. Groups are CTRL (control group), PE (paw edema group), DKP (dexketoprofen group), and NanoDKP: (the optimal dexketoprofen ZN formulation group) (significant at *p* < 0.05).

The effects of F7 on formalin-induced paw edema inflammation in rats were investigated. As depicted in Figure 5, no significant difference was observed in p-values for paw edema at the time of formalin injection compared to other groups. Paw edema was significantly induced at 1-, 2-, 4-, and 24-h post-injection, with notable significance at 4 h (*p* < 0.0001), indicating the establishment of formalin-induced inflammation. These results are consistent with previous studies (Soyocak et al., 2019; Lee and Jeong, 2002), which reported that formalin administration resulted in swelling of the paws and an increase in vascular permeability. Group III (DKP group), which received the drug only, showed a mild ameliorative effect on the inflammation (*p* < 0.05) compared to group II (PE group), while group IV (DKP Nano group), which received the optimal ZN formulation (F7), demonstrated a markedly greater reduction in inflammation (*p* < 0.001), with no significant difference from the control group.

**FIGURE 6:**
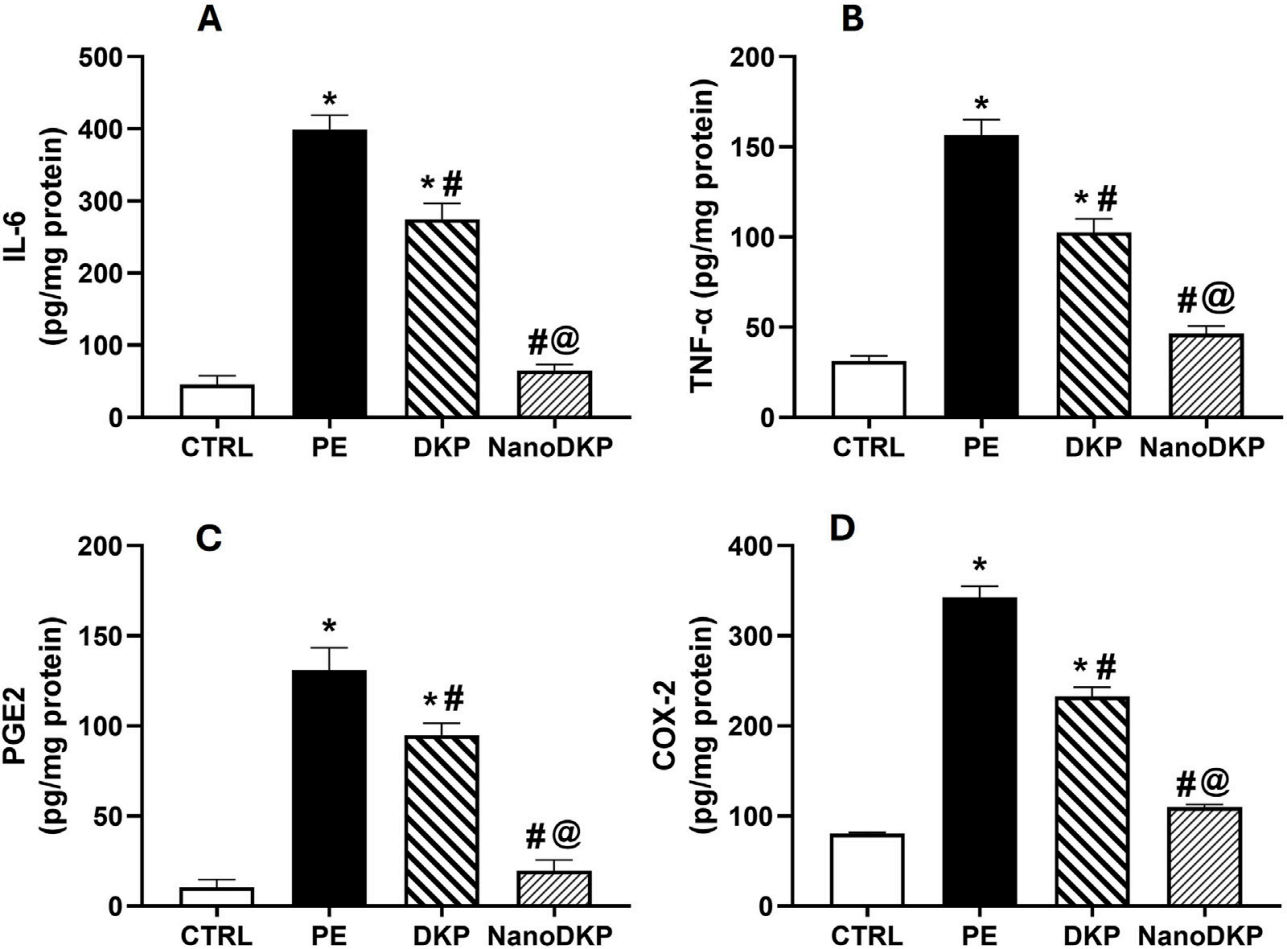
Effect of F7 on the level of inflammatory cytokines in rats with foot swelling. **(A)** IL-6, **(B)** TNF-α, **(C)** PGE2, and **(D)** COX2. Data are represented as the mean ± SD; *, #, and @ p < 0.05 compared to CRTL, PE, and DKP groups, respectively, using one-way ANOVA, followed by Tukey’s multiple comparisons test. CTRL, control group; PE, paw edema group; DKP, dexketoprofen group; and NanoDKP, the optimal dexketoprofen ZN formulation group.

TNF-α, IL-6, PGE2, and COX-2 are pivotal mediators in inflammatory paw edema, each contributing to distinct mechanistic pathways. TNF-α drives edema formation by enhancing vascular permeability and neutrophil migration via p55 receptor activation, as demonstrated in carrageenan-induced edema models where TNF-α neutralization or receptor knockout significantly reduced edema and mechanical hypersensitivity (Rocha et al., 2006; Mansouri et al., 2015). IL-6 exhibits systemic pro-inflammatory effects, with serum levels directly correlating with paw swelling kinetics in adjuvant arthritis, while pharmacological inhibition of IL-6 pathways ameliorates both edema and cytokine activity (Leisten et al., 1990; Karim et al., 2019). PGE2, synthesized through COX-2 upregulation, mediates vasodilation and nociception via EP3 receptor-dependent signaling cascades involving phospholipase C and protein kinase C (Claudino et al., 2006; Damas and Liégeois, 1999). The critical role of COX-2 in sustaining PGE2 production is highlighted by its selective inhibition, which rapidly normalizes prostaglandin levels and resolves edema (Karim et al., 2019; Anderson et al., 1996). These mediators collectively represent interconnected facets of inflammation—TNF-α and IL-6 modulate immune cell recruitment and cytokine amplification, while the COX-2/PGE2 axis regulates vascular dynamics and pain sensitization. Targeting these pathways through dexketoprofen-loaded zein nanoparticles likely achieves synergistic anti-inflammatory effects by suppressing COX-2-mediated prostaglandin synthesis (Jori et al., 2024) and attenuating cytokine-driven neutrophil infiltration (Mansouri et al., 2015), thereby disrupting the inflammatory cascade at multiple nodes. As illustrated in Figure 6, the levels of inflammatory cytokines IL-6, TNF-α, PGE2, and COX2 were assessed using the ELISA technique to verify the anti-inflammatory effect of dexketoprofen. Twenty-four hours after formalin injection, there was a significant increase in IL-6 [F (3, 20) = 631.9, *p* < 0.0001], TNF-α [F (3, 20) = 84.61, *p* < 0.0001], PGE2 [F (3, 20) = 312.8, *p* < 0.0001], and COX2 [F (3, 20) = 216.1, *p* < 0.0001] by 8.7-, 5.4-, 13-, and 4.3-fold, respectively, compared to the control group. The prior administration of dexketoprofen significantly mitigated the formalin-induced elevation in these inflammatory markers by 31%, 34%, 27.5%, and 32%, respectively, compared to the formalin group. Notably, F7 exhibited a marked anti-inflammatory effect, restoring the inflammatory markers to near-normal levels by decreasing IL-6, TNF-α, PGE2, and COX2 levels by 76%, 55%, 79%, and 53%, respectively, compared to the DKP group.

**FIGURE 7:**
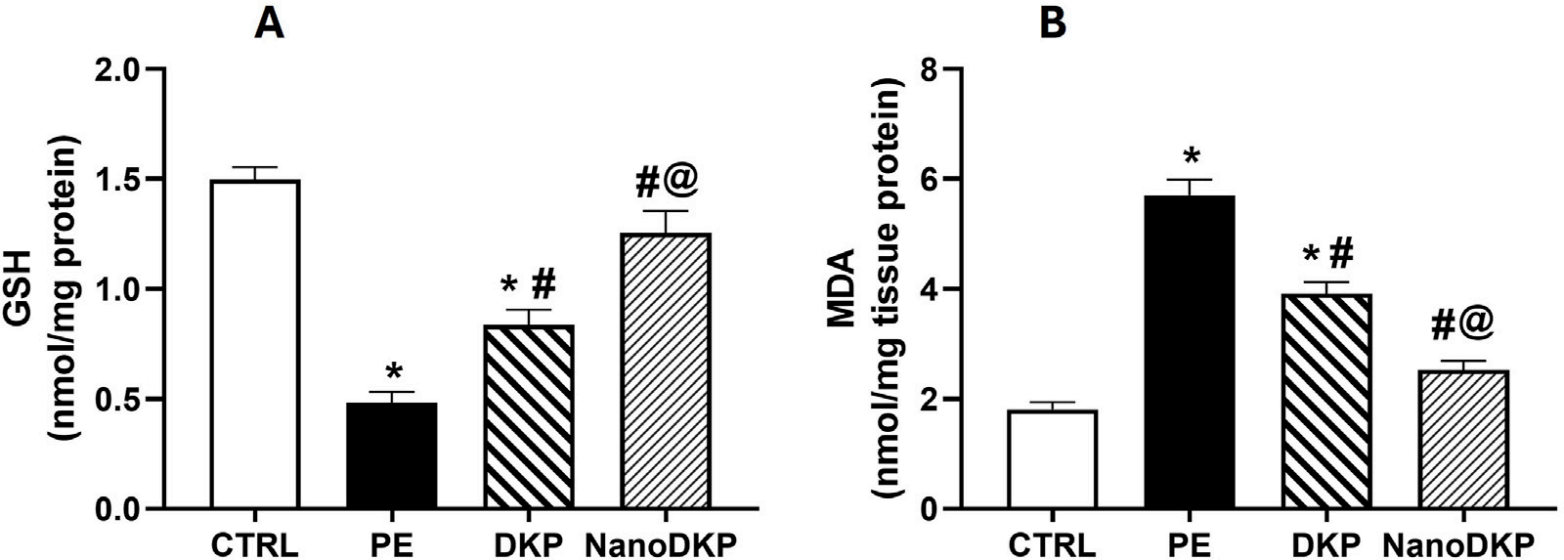
Effect of F7 on the level of oxidative stress biomarkers in rats with foot swelling. **(A)** GSH and **(B)** MDA. Data are represented as the mean ± SD; *, #, and @ *p* < 0.05 compared to control, PE, and DKP groups, respectively, using one-way ANOVA, followed by Tukey’s multiple comparisons test. CTRL, control group; PE, paw edema group; DKP, dexketoprofen group; and NanoDKP, the optimal dexketoprofen ZN formulation group.

MDA and GSH are critical biomarkers of oxidative stress-mediated inflammation in paw edema, offering complementary insights into redox balance and tissue damage. MDA, a terminal product of lipid peroxidation, directly reflects ROS-induced membrane damage during inflammation. Its accumulation triggers pro-inflammatory signaling by forming cytotoxic adducts with macromolecules, activating NF-κB-driven pathways that amplify cytokine production (e.g., TNF-α and IL-6) and neutrophil infiltration, exacerbating edema (Zhang et al., 2020). For instance, in carrageenan-induced edema, elevated MDA correlates with increased vascular leakage and hyperalgesia, while antioxidant therapies that reduce MDA concurrently suppress inflammation (Zhang et al., 2020). Conversely, GSH, the primary intracellular antioxidant, counteracts oxidative stress by scavenging ROS and maintaining cellular redox homeostasis. Its depletion disrupts PTEN/PI3K/AKT signaling, leading to unchecked ROS production and heightened pro-inflammatory cytokine release (e.g., TNF-α and IL-1β), which perpetuates edema and tissue injury (Albarakati, 2022). Studies on adjuvant-induced arthritis show that GSH replenishment attenuates edema by restoring redox balance and suppressing NF-κB activation. The findings of our study align with these previous reports, showing that the administration of formalin significantly elevated oxidative stress damage, as indicated by a 3.2-fold increase in MDA levels [F (3, 20) = 69.61, *p* < 0.0001] and a 64% reduction in GSH levels [F (3, 20) = 38.59, *p* < 0.0001] compared to the control group (Figure 7). However, pre-treatment with dexketoprofen effectively mitigated these detrimental effects, resulting in a 32% reduction in MDA levels (*p* < 0.0107) and a 1.7-fold increase in GSH levels (*p* < 0.0001) relative to the formalin group. Notably, treatment with the F7 (NanoDKP) formulation further enhanced the antioxidant response, producing a 36% reduction in MDA levels (p = 0.0006) and a 49% increase in GSH levels (p = 0.0028) compared to the DKP group. These values were statistically indistinguishable from those of the control group (p > 0.05), indicating that NanoDKP normalized oxidative stress markers to near-baseline levels.

**FIGURE 8:**
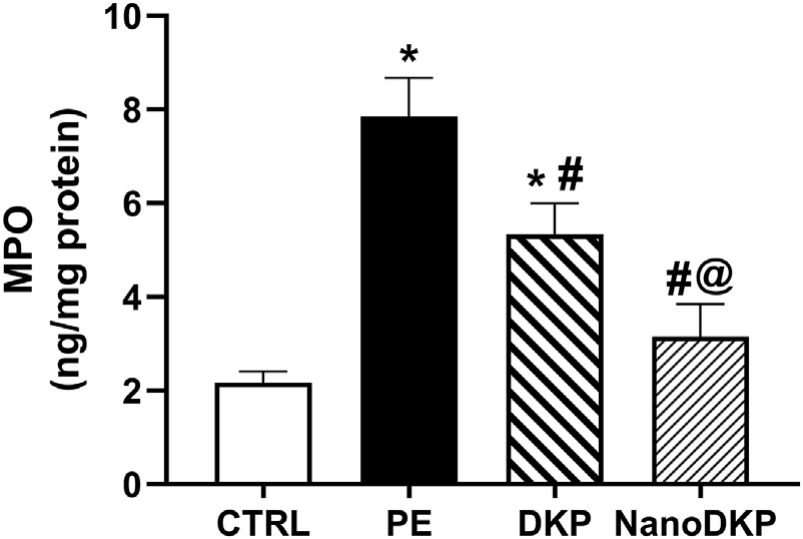
Effect of F7 on the activity of MPO biomarkers in rats with foot swelling. Data are represented as the mean ± SD; *, #, and @ *p* < 0.05 compared to control, PE, and DKP groups, respectively, using one-way ANOVA, followed by Tukey’s multiple comparisons test. CTRL, control group; PE, paw edema group; DKP, dexketoprofen group; and NanoDKP, the optimal dexketoprofen ZN formulation group.

MPO is a critical mediator of neutrophil-driven inflammation in paw edema, serving as both a biomarker of neutrophil infiltration and a direct contributor to oxidative tissue damage. Released by activated neutrophils, MPO catalyzes the production of hypochlorous acid (HOCl) and other reactive oxygen species (ROS), exacerbating oxidative stress and lipid peroxidation in inflamed tissues (Soyocak et al., 2019). Elevated MPO activity correlates strongly with the severity of inflammation, as demonstrated in carrageenan-induced paw edema models where MPO levels surge alongside neutrophil recruitment and edema formation (Soyocak et al., 2019). Pharmacological interventions, such as tannic acid and memantine, attenuate paw swelling by suppressing MPO activity, thereby reducing ROS generation and subsequent lipid peroxidation (e.g., MDA levels) (Soyocak et al., 2019; Azarbaijani et al., 2021). MPO’s role extends beyond oxidative damage; it amplifies pro-inflammatory signaling by promoting cytokine release and endothelial activation, creating a feedback loop that sustains neutrophil influx (Puttaswamy et al., 2018; Kothari et al., 2011). In formalin-induced edema, MPO inhibition coincides with diminished vascular permeability and pain sensitization, underscoring its dual role in inflammation and tissue injury (Soyocak et al., 2019). In accordance, the MPO activity in paw tissue was elevated by 3.7-fold 24 h after formalin injection compared to the control group [F (3, 20) = 91.58, *p* < 0.0001]. As illustrated in Figure 8, pre-treatment with dexketoprofen significantly reduced the increase in MPO activity in formalin-treated paw tissue by 32% (*p* < 0.0001). Notably, treatment with dexketoprofen ZNs resulted in a significant 41% reduction in MPO activity (*p* < 0.0001) compared to the DKP group, nearly restoring levels to those observed in the control group.

#### Histopathological examination

**FIGURE 9:**
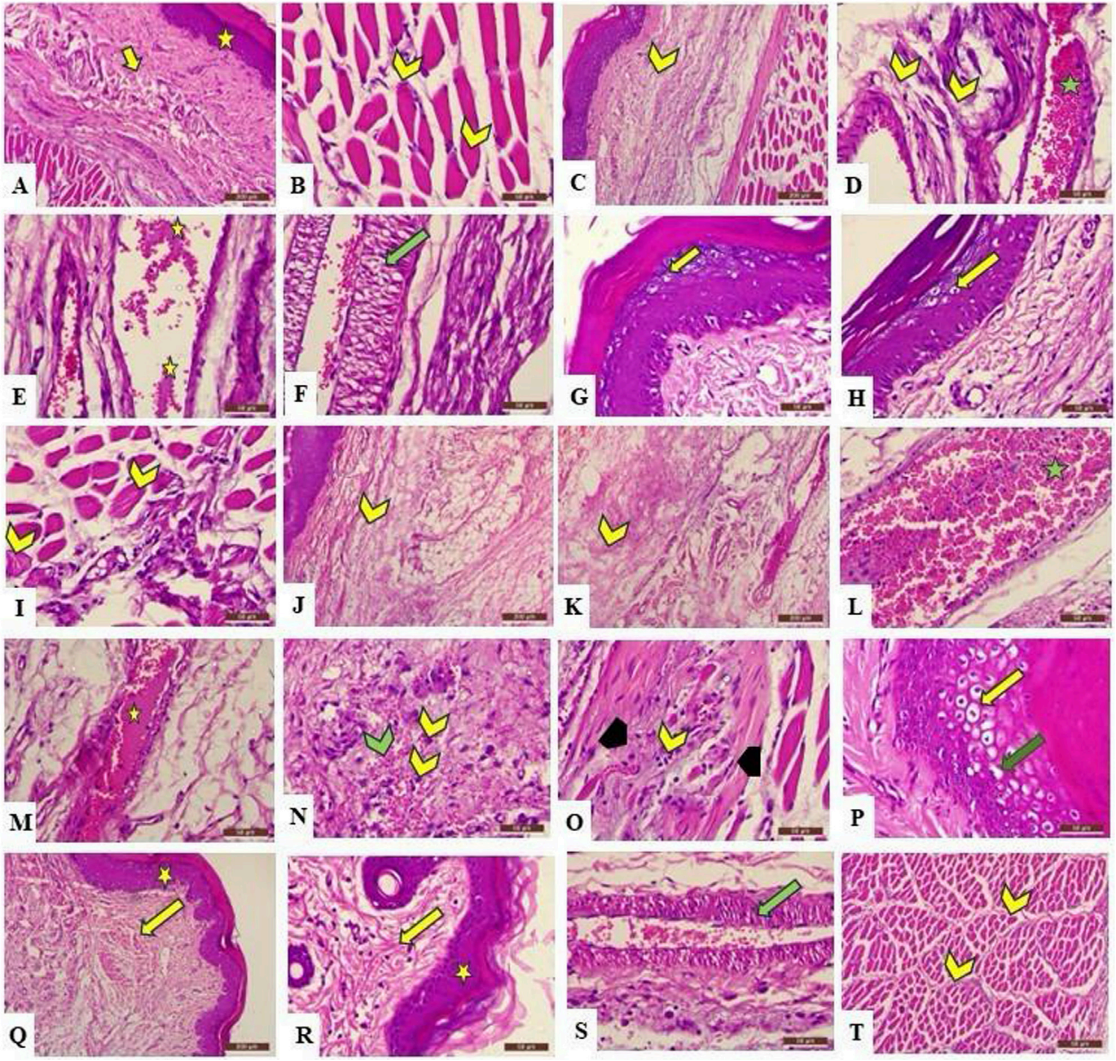
Photomicrograph of the H&E-stained section of rat’s skin showing A (X100) and B (X400): the control group reveals normal histological architecture of the epidermis **(A)** (star) and dermis **(A)** (arrow), which exhibits a normal arrangement of collagen fibers, in addition to normal striated muscle fibers under the skin **(B)** (chevron) that show peripheral nuclei in cross section. **(C)** (X100) and **(D–I)** (X400): Group 2 exhibits deterioration of the tissue in the form of dispersion of the collagen fibers in the dermis because of dermal edema **(C,D)** (yellow chevron). Moreover, blood vessels appear congested and engorged with blood **(D)** (green star), and edematous fluid is present **(E)** (yellow star), with vacuolation of the tunica media **(F)** (green arrow). In addition, ballooning degeneration and acute cellular swelling of keratinocytes are also observed **(G,H)** (yellow arrow). The same group also reveals accumulation of inflammatory cells between muscle fibers, which have lost their normal arrangement **(I)** (yellow chevron). **(J,K)** (X100) and **(M–Q)** (X400): Group 3 shows the disintegration of collagen fibers by edematous fluid **(J,K)** (yellow chevron), blood vessel congestion **(L)** (green star), edema **(M)** (yellow star), hemorrhage **(N)** (green chevron), and infiltration of inflammatory cells **(N)** (yellow chevron) in the dermis, in addition to the deterioration of the striated muscle fibers **(O)** (black arrowhead), which appear invaded by inflammatory cells **(O)** (yellow chevron). Moreover, ballooning degeneration **(P)** (yellow arrow) and acute cellular swelling **(P)** (green arrow) of the stratum spongiosum are observed. **(Q)** (X100) and **(R–T)** (X400): Group 4 exhibits normal microscopical structure of the epidermis **(Q,R)** (yellow star) and the dermis **(Q,R)** (yellow arrow), indicating no inflammatory changes, with less vacuolation of the tunica intima of blood vessel **(S)**; (green arrow) and normal arrangement of striated muscle bundles surrounded by perimysium **(T)** (yellow chevron).

H&E-stained skin tissues from all groups were examined using light microscopy with different magnification powers. Tissues obtained from the control group (group I) revealed the normal structure of the stratified squamous keratinized epithelium of the epidermis and normal histological architecture of the dense irregular connective tissue of the dermis and dermal glands, in addition to the normal arrangement of the striated muscle fibers under the skin layers. On the other hand, group II exhibited different inflammatory changes in the skin tissue in the form of disintegration of collagen fibers of the dermis by edema, congestion of dermal blood vessels that appeared engorged with blood and edematous fluid, and vacuolation of tunica media. Moreover, keratinocytes of the epidermal layer, particularly cells of the stratum spinosum, revealed ballooning degeneration and acute cellular swelling. The striated muscle appeared deteriorated and was invaded by inflammatory cells. The previously mentioned changes were also observed in group III, which showed inflammatory changes in the dermis in the form of deteriorated collagen fibers separated by edematous fluid, along with congested blood vessels exhibiting edema and engorgement with blood. Moreover, hemorrhage and inflammatory cell infiltration of the dermal tissue were also detected. Ballooning degeneration of stratum spinosum cells that appeared swollen and pale was clearly revealed. Interestingly, the group treated with F7 (group IV) exhibited an improvement in the destructed tissue with mild inflammatory changes that exhibited mild vacuolation of the tunica media of dermal blood vessels, with normal stratified squamous keratinized epithelium of the epidermis and other structures of the dermis. Furthermore, the striated muscle of group IV showed the regular arrangement of normal striated muscle bundles surrounded by connective tissue fibers of the perimysium (Figure 9).

#### Limitation

This study used a prophylactic model, administering the NanoDKP formulation prior to formalin-induced edema, to assess its ability to modulate early inflammatory mediators (e.g., prostaglandins and histamine) and establish proof-of-concept efficacy. Although this design aligns with preclinical studies evaluating preventive strategies for anticipated inflammation (e.g., post-surgical settings), we acknowledge that therapeutic models (post-induction administration) are needed to fully extrapolate findings to clinical scenarios involving established inflammation. Future studies will explore these therapeutic contexts.

## Conclusion

This research involved the preparation of ZNs as carriers for dexketoprofen for the treatment of inflammation. Dexketoprofen-loaded ZNs were produced by applying the ethanol injection process. A 2^3^ full-factorial design was used to assess the results and conclude the optimal ZN formulation. The *in silico* study of the optimal formulation highlighted the strong binding affinity of zein for dexketoprofen. The optimal formulation had spherical morphology, high EE%, and small PS. The *in vivo* investigation of the optimal dexketoprofen-loaded ZNs has shown a more pronounced anti-inflammatory activity against formalin-induced paw edema than the drug, along with normal histological results. The optimal ZN formulation also exhibited a significant reduction in TNF-α, COX-2, IL-6, PGE2, and MDA levels while significantly increasing GSH levels compared to dexketoprofen. Concisely, the overall conclusion is that ZNs are a promising, effective, and convenient system for the topical delivery of dexketoprofen for the treatment of inflammation.

## Data Availability

The original contributions presented in the study are included in the article/supplementary material; further inquiries can be directed to the corresponding authors.
