# Correction: Formulation of zein nanoparticles for augmenting the anti-inflammatory activity of dexketoprofen

**DOI:** 10.3389/fphar.2025.1658349

**Published:** 2025-08-07

**Authors:** Mahmoud A. El Hassab, Mona H. Ibrahim, Sherif S. Abdel Mageed, Abdulla M. A. Mahmoud, Zainab Sabry Othman Ahmed, Shaimaa Mosallam, Manar Adel Abdelbari, Eman F. Khaleel, Maram A. El Hasab, Asmaa Elsayyad, Shady Allam, Moaz A. Eltabeeb, Ahmed T. Negmeldin, Wagdy M. Eldehna, Rofida Albash

**Affiliations:** ^1^ Department of Medicinal Chemistry, Faculty of Pharmacy, King Salaman International University (KSIU), South Sinai, Egypt; ^2^ Department of Pharmaceutical Medicinal Chemistry and Drug Design, Faculty of Pharmacy (Girls), Al-Azhar University, Cairo, Egypt; ^3^ Pharmacology and Toxicology Department, Faculty of Pharmacy, Badr University in Cairo (BUC), Cairo, Egypt; ^4^ Department of Cytology and Histology, Faculty of Veterinary Medicine, Cairo University, Giza, Egypt; ^5^ Faculty of Veterinary Medicine, King Salman International University, South Sinai, Egypt; ^6^ Department of Pharmaceutics and Industrial Pharmacy, Faculty of Pharmacy, October 6 University, Giza, Egypt; ^7^ Department of Medical Physiology, College of Medicine, King Khalid University, Asir, Saudi Arabia; ^8^ Department of Pediatrics, Faculty of Medicine, Tanta University, Tanta, Egypt; ^9^ Department of Pharmacology, Faculty of Veterinary Medicine, Mansoura University, Mansoura, Egypt; ^10^ Department of Physiology, and Pharmacology, Faculty of Veterinary Medicine, King Salman International University, South Sinai, Egypt; ^11^ Department of Pharmacology and Toxicology, Faculty of Pharmacy, Menoufia University, Menoufia, Egypt; ^12^ Department of Pharmacology and Toxicology, Faculty of Pharmacy, Menoufia National University, Menoufia, Egypt; ^13^ Department of Industrial Pharmacy, College of Pharmaceutical Sciences and Drug Manufacturing, Misr University for Science and Technology, Giza, Egypt; ^14^ Department of Pharmaceutical Sciences, College of Pharmacy and Thumbay Research Institute for Precision Medicine, Gulf Medical University, Ajman, United Arab Emirates; ^15^ Department of Pharmaceutical Organic Chemistry, Faculty of Pharmacy, Cairo University, Cairo, Egypt; ^16^ Department of Pharmaceutical Chemistry, Faculty of Pharmacy, Kafrelsheikh University, Kafrelsheikh, Egypt; ^17^ Department of Pharmaceutics, College of Pharmaceutical Sciences and Drug Manufacturing, Misr University for Science and Technology, Giza, Egypt

**Keywords:** dexketoprofen, zein nanoparticles, inflammation, formalin-induced paw edema, topical delivery, docking

**Affiliation 12** (assigned to **Shady Allam**) was erroneously given as “Department of Pharmaceutics, College of Pharmaceutical Sciences and Drug Manufacturing, Misr University for Science and Technology, Giza, Egypt.” This should have been **Affiliation 17**. **Affiliation 12** has been updated as “Department of Pharmacology and Toxicology, Faculty of Pharmacy, Menoufia National University, Menoufia, Egypt.”

**Affiliation 17** was omitted for author **Rofida Albash**, who was erroneously assigned **Affiliation 12**. This affiliation (“Department of Pharmaceutics, College of Pharmaceutical Sciences and Drug Manufacturing, Misr University for Science and Technology, Giza, Egypt”) has now been added for author **Rofida Albash** in place of **Affiliation 12**.

The original article has been updated.

